# Public Medical Appeals and Government Online Responses: Big Data Analysis Based on Chinese Digital Governance Platforms

**DOI:** 10.2196/70087

**Published:** 2025-08-06

**Authors:** Hebin Li, Zhihan Liu, Ziyan Zhang, Lu Ping, Wenxin Gu, Yuan Yao

**Affiliations:** 1 School of Public Administration, Central South University, 20 Shaoshan South Road, Tianxin District, Changsha, 410004, China, 86 0731-88876916; 2 Business School, Central South University, Changsha, China

**Keywords:** medical appeals, government online responses, digital governance, content analysis, co-word analysis, logistic regression

## Abstract

**Background:**

In the era of internet-based governance, online public appeals—particularly those related to health care—have emerged as a crucial channel through which citizens articulate their needs and concerns.

**Objective:**

This study aims to investigate the thematic structure, emotional tone, and underlying logic of governmental responses related to public medical appeals in China.

**Methods:**

We collected messages posted on the “Message Board for Leaders” hosted by People’s Daily Online between January 2022 and November 2023 to identify valid medical appeals for analysis. (1) Key themes of public appeals were identified using the term frequency-inverse document frequency model for feature word extraction, followed by hierarchical cluster analysis. (2) Sentiment classification was conducted using supervised machine learning, with additional validation through sentiment scores derived from a lexicon-based approach. (3) A binary logistic regression model was employed to examine the influence of textual, transactional, and macro-environmental factors on the likelihood of receiving a government response. Robustness was tested using a Probit model.

**Results:**

From a total of 404,428 online appeals, 8864 valid public medical messages were retained after filtering. These primarily concerned pandemic control, fertility policies, health care institutions, and insurance issues. Negative sentiment predominated across message types, accounting for 3328 out of 3877 (85.84%) complaints/help-seeking messages, 1666 out of 2381 (69.97%) consultation messages, and 1710 out of 2606 (65.62%) suggestions. Regression analysis revealed that textual features, issue complexity, and benefit attribution were not significantly associated with government responsiveness. Specifically, for textual features, taking the epidemic issue as the reference category in the appeal theme, the *P* values were as follows: fertility issue (*P*=.63), hospital issue (*P*=.63), security issue (*P*=.72), and other issues (*P*=.34). Other textual features include appeal content (*P*=.80), appeal sentiment (*P*=.64), and appeal title (*P*=.55). Regarding the difficulty of resolving incidents, with low difficulty as the reference category, the *P* values were moderate difficulty (*P*=.59) and high difficulty (*P*=.96). For benefits attribution, using individual interest as the reference, collective interest (*P=.*25) was not statistically significant*.* By contrast, macro-level factors—specifically internet penetration, education, economic development, and labor union strength—had significant effects. Compared with areas with lower levels, higher internet penetration (odds ratio 1.577-9.930, *P*=.004 to <.001), education (odds ratio 2.497, *P*<.001), and gross domestic product (odds ratio 2.599, *P<.*001) were associated with increased responsiveness. Conversely, medium (odds ratio 0.565, *P*<.001) and high (odds ratio 0.116, *P*<.001) levels of labor union development were linked to lower response odds.

**Conclusions:**

Public medical appeals exhibit 5 defining characteristics: urgency induced by pandemic conditions, connections to fertility policy reforms, tensions between the efficacy and costs of medical services, challenges related to cross-regional insurance coverage, and a predominance of negative sentiment. The findings indicate that textual features and issue-specific content exert limited influence on government responsiveness, likely due to the politically sensitive and complex nature of health care**–**related topics. Instead, macro-level environmental factors emerge as key determinants. These insights can inform the optimization of response mechanisms on digital health platforms and offer valuable theoretical and empirical contributions to the advancement of health information dissemination and digital governance within the health care sector.

## Introduction

### Background

In recent years, the deep integration of digital technologies with health care has brought about significant transformations in the health care landscape. Digital health tools such as electronic health records, telemedicine, and mobile health (mHealth) have not only reshaped the delivery paradigm of modern medical services but have also revolutionized modes of health information exchange [1]. Against the backdrop of widespread digitalization in the health sector, government-operated digital health platforms have emerged as increasingly important sources of health-related information [2,3]. Citizens now use these platforms to express their medical appeals, facilitating physician-patient communication [4] and promoting interactive governance in health care, thereby supporting the development of digital health governance.

Given the complexity, sensitivity, and public welfare implications of the health care sector, it has become a core domain for public appeals. During the process of receiving health care services, individuals—motivated by the protection of health rights, a desire to improve service quality, or the need for policy adjustments—frequently submit collective or individual suggestions and demands to medical institutions and health authorities [5]. In response, governments reply to public appeals on digital platforms and often take active measures to resolve the raised issues [6,7]. A representative example is the *Message Board for Leaders* (*MBL*), operated by the People’s Daily Online (*人民网*), a flagship official e-governance platform in China that enables direct public communication with national ministries and local party-government leaders. Increasingly, the public is using such platforms to consult on health care policies or voice specific medical concerns, making this an important channel for government-citizen interaction [8].

Despite the growing use of government digital platforms for expressing health care–related concerns, existing research presents several limitations. First, much of the literature focuses on the macro-architecture of online government responsiveness [9] or universal mechanisms [10], with limited attention to health care as a distinct domain of governance. Second, studies examining government responses in the health sector have largely concentrated on emergency responses to public health crises, while investigations into routine medical appeals remain scarce [7,11]. Third, the theoretical frameworks employed are often fragmented and lack a coherent mechanism to explain how governments respond to public appeals within the healthcare context.

To address these gaps, this study draws on textual data from the health care section of the People’s Daily Online’s MBL. Using text mining techniques, we identify the dominant themes and emotional characteristics of public medical appeals. By incorporating both textual and nontextual factors into our research hypotheses, we systematically explore the mechanisms influencing government responsiveness to these appeals. Ultimately, this research aims to elucidate the thematic patterns, emotional dynamics, and response logics of public medical appeals within the health care domain, thereby offering policy-relevant insights to improve government responsiveness and service quality in the era of digital health governance.

### Research Hypotheses

Public medical appeals are often characterized by high issue complexity, substantial societal implications, and intense emotional expression. Research in health communication suggests that features such as the length of the appeal, emotional intensity, and message clarity play critical roles in shaping the dissemination and interpretation of health-related information [12]. Government responsiveness in this sector goes beyond merely interpreting policy texts—it must also address tangible problems in medical service delivery. Furthermore, macro-level environmental factors, such as economic development, educational attainment, digital infrastructure, and labor union development, significantly influence the effectiveness of health information governance [13]. Despite these insights, prior studies have largely examined these factors in isolation, leading to a fragmented understanding of government responsiveness that fails to capture the interplay between textual and contextual variables.

To address this gap, this study proposes research hypotheses that incorporate both textual (eg, themes, sentiment, content, title) and nontextual (eg, difficulty of resolving incidents, benefits attribution, regional socioeconomic status) factors to comprehensively examine the determinants of government responsiveness on online platforms. This integrative analytical approach enables an examination of how different types of citizen demands interact with contextual realities to shape online governance outcomes. Given the unique political sensitivity and public salience of the health care domain, these research hypotheses also offer important implications and references for future empirical studies and theoretical exploration in the field of digital health governance. The study extends existing work in health informatics by incorporating governance and institutional dynamics into the study of digital health communication.

### Textual Factors and Government Responsiveness

The decentralized and symbolic nature of online governance platforms has diminished the influence of traditional hierarchical elements, thereby elevating the role of discursive expression in shaping governmental responses to public concerns [14,15]. Prior research has examined how various textual characteristics of citizen appeals affect patterns of government responsiveness [16-19]. Subtle differences in textual content and distinctive narrative features may significantly influence the immediate reactions of information recipients [20]. Therefore, we propose the following main hypothesis:

- *H1:Textual factors significantly influence government responsiveness.*

First, public appeals are typically organized around specific themes, and government responses tend to exhibit selectivity and domain-specific preferences. Issues such as public health emergencies are more likely to trigger prompt and high-quality governmental responses due to their urgency and political sensitivity. This leads to the first subhypothesis:

- *H1a: Variations in the themes of appeals influence the likelihood of government response*.

Second, the increasing complexity and multidimensionality of public medical appeals represent a defining feature of online discourse. In general, more complex appeals tend to provide richer contextual information and greater detail, which may enhance their perceived legitimacy and urgency [16,21]. As a result, such appeals are more likely to attract government attention and elicit a response:

- *H1b: The complexity of appeal content positively affects government responsiveness.*

Third, emotional intensity embedded in public appeals can serve as a rhetorical strategy to pressure authorities into responding [22]. For instance, a prior study in China found that using threat-based language, such as referencing higher-level complaints or collective actions, increased the likelihood of receiving a government response [23]. Emotional contagion in appeals can also amplify visibility and urgency [24]:

- *H1c: The emotional intensity of appeals influences the likelihood of government response*.

Finally, the structure of appeal titles serves as an initial signal that shapes government perception, priority-setting, and response behavior on digital governance platforms. Overly brief titles may lack essential contextual cues, whereas excessively long titles can create cognitive overload, delaying, or even obstructing response decisions [25]:

- *H1d: The structural characteristics of appeal titles, particularly text length, affect government responsiveness*.

### Transactional Factors and Government Responsiveness

Beyond textual characteristics, the intrinsic attributes of the public issue itself—referred to here as transactional factors—may exert a critical influence on government response behavior. Public affairs vary significantly in their policy salience, administrative priority, and procedural complexity within government systems. These variations subtly but meaningfully shape governmental decisions about whether and how to respond. Prior studies have demonstrated that problem-solving difficulty [26] and the nature of stakeholder interests [27] can alter online response strategies. Accordingly, we propose the following hypothesis:

- *H2: Transactional factors significantly influence government responsiveness*.

First, the degree of difficulty associated with addressing a particular issue affects how quickly and substantively the government is likely to respond. Appeals involving complex, cross-sectoral, or highly sensitive problems often require extensive administrative coordination and carry political risks. By contrast, less complex appeals may be resolved more readily and are more likely to receive prompt replies [28]:

- *H2a: Appeals involving lower problem-solving difficulty are more likely to elicit a government response*.

Second, the nature of stakeholder interests reflected in the appeal may influence the government’s prioritization. Appeals framed around collective interests, such as those that concern communities, groups, or multiple beneficiaries, tend to attract greater governmental attention. This is because mishandling such issues may trigger broader dissatisfaction or even public unrest. Compared with individually framed complaints, collective appeals are therefore more likely to receive substantive and careful responses [21,29,30]:

- *H2b: Appeals involving collective interests are more likely to receive a government response than those based on individual interests*.

### Macro-Environmental Factors and Government Responsiveness

Discursive practices are inherently embedded in specific temporal and spatial contexts. Government responsiveness, in this regard, represents an administrative system’s output in reaction to environmental stimuli, following an “input-output-feedback” logic under particular institutional conditions [31]. Macro-level factors, such as economic development [32], technological infrastructure [33], educational attainment [34], and labor union development, serve as exogenous enablers that shape both the frequency of public engagement and the structural capacity of local governments to process and respond to digital appeals. Based on this reasoning, we propose the following overarching hypothesis:

- *H3: Macro-environmental factors significantly influence government responsiveness*.

First, education level may impact how individuals understand public issues and utilize social resources to engage with governmental institutions. Higher educational attainment tends to be associated with enhanced political awareness, improved articulation of appeals, and more proactive digital participation [13,35]. These factors, in turn, may prompt more frequent and substantive government responses:

- *H3a: Regions with higher educational attainment are more likely to receive government responses to public medical appeals*.

Second, internet penetration is a crucial determinant of the government’s digital responsiveness. On the one hand, advances in digital communication technologies reduce information asymmetries between the public and government agencies, enabling more efficient civic expression [36]. On the other hand, the rapid diffusion of information online increases the potential for public unrest or collective action [37,38], thereby incentivizing local governments to proactively address digital appeals as a risk mitigation strategy:

- *H3b: Regions with higher internet penetration are more likely to receive government responses to public medical appeals*.

Third, local economic development provides the material foundation for digital government responsiveness. Stronger fiscal capacity enables investment in e-government infrastructure, human resources, technological tools, and system maintenance [39,40]. Moreover, wealthier regions tend to have more engaged citizenries, stronger civic expectations [41], and more intensive scrutiny of governmental performance, which collectively pressure governments to be more responsive:

- *H3c: Regions with higher levels of economic development are more likely to receive government responses to public medical appeals*.

Finally, unlike labor unions in Western contexts, which primarily function to protect members’ rights and often express public appeals through demonstrations or collective actions [42], labor unions in China typically possess semiofficial characteristics and serve as extensions of governmental functions. These organizations frequently act as quasi-governance actors [43]. When faced with public medical appeals, Chinese labor unions often engage in internal mediation, absorbing and resolving certain demands through internal channels. This mediatory role helps alleviate the direct pressure on the government to respond to such appeals. Thus, despite institutional and operational differences between Chinese and Western labor unions, the former tend to assume a *mediator* role rather than an *adversarial* one. Accordingly, labor union development appears to exert a significant influence on government responsiveness. Based on this logic, we propose the following hypothesis:

- *H3d: Regions with higher levels of labor union development are less likely to receive government responses to public medical appeals*.

## Methods

### Ethics Considerations

This study is a secondary analysis of publicly available and anonymized data obtained from the MBL platform hosted by People’s Daily Online. The dataset contains no personally identifiable information or sensitive user metadata. All messages were publicly posted and do not include real names or direct identifiers. The researchers did not collect data directly or interact with the individuals who submitted the messages. Given that the data are fully anonymized and publicly accessible, and that no intervention, interaction, or identifiable private information is involved, this study does not constitute human participant research as defined by most institutional policies. Accordingly, ethical review and informed consent were not required.

### Overview

This study adopts a mixed-methods approach combining content analysis, co-word analysis, and logistic regression to investigate public medical appeals submitted via Chinese digital governance platforms. Content analysis is used to interpret social meaning by examining the frequency, sentiment, and contextual usage of specific words or concepts within the appeals, enabling the identification of discursive patterns and emotional tendencies [44]. Co-word analysis is used to uncover the thematic structure of public appeals by analyzing the co-occurrence relationships among keywords, thereby delineating the major areas of citizen concern in the health care domain [45]. To assess the influence of various explanatory variables on government responsiveness, logistic regression is applied. This statistical model estimates the likelihood of government responses based on textual (eg, sentiment, appeal themes) and nontextual (eg, socioeconomic conditions) factors.

### Data Collection

The data analyzed in this study were sourced from the *MBL* platform hosted by People's Daily Online. This platform was selected as the data source for the reasons mentioned below.

First, in terms of representativeness, the MBL is currently the only nationwide platform in China that facilitates direct online communication between citizens and government officials. Covering all 31 provinces and most major municipalities, the platform offers a highly representative and diverse dataset that reflects the breadth of regional governance dynamics and public concerns.

Second, the platform’s structure and content are particularly well-suited to the research objectives of this study, namely, extracting thematic patterns, analyzing sentiment, and identifying response determinants. The MBL allows users to submit personalized appeals to specific government departments or officials, and health care consistently ranks among the top 10 domains of public concern on the platform. The richness and clarity of the text-based appeal records, along with associated response data, provide a robust empirical foundation for computational analysis.

As a result of the platform’s technical limitation in displaying only recent historical data (generally up to around January of the previous year), data collection was carried out in December 2023 using the *HouYi Collector*, a publicly available web scraping tool developed by a former Google Search engineering team. The final dataset includes public medical appeals submitted between January 2022 and November 2023, ensuring both temporal relevance and analytical completeness.

### Data Preprocessing

The data cleaning and preprocessing process of this study consisted of 2 primary stages, systematically conducted by the research team.

The initial phase of data preparation involved raw data extraction and filtering. Two researchers with expertise in health informatics systematically collected all publicly available messages from the MBL platform, focusing on those addressed to provincial and capital city officials across China. Data scraping was performed using HouYi Collector, a web crawler tool, yielding a total of 404,428 message records spanning a wide range of public concerns.

Subsequently, a manual screening process was conducted to identify messages specifically related to public health care issues. This yielded 10,800 potentially relevant entries. Alongside the full text of both the public messages and corresponding government replies, structured metadata—such as message titles, categories, submission types, processing statuses, designated government departments, and time stamps—were extracted for further analysis.

Following professional review and the application of predefined inclusion criteria, a final dataset comprising 8864 valid records was retained. The inclusion criteria were as follows:

- Messages had to be directly related to public health care concerns; generic complaints or background narratives unrelated to health services were excluded.
- Entries were required to demonstrate basic linguistic integrity; records containing garbled characters, blank content, or text consisting solely of emoticons were discarded.
- Only appeals submitted by verified users were retained, whereas commercial advertisements and clearly fabricated or bot-generated content were removed.
- In cases where a single user submitted multiple entries within a short time frame, only the most representative message was preserved to minimize sampling bias.

This curated dataset served as the foundation for all subsequent analyses, including topic classification, sentiment analysis, and logistic regression modeling.

In the second phase, preprocessing was applied to the list of feature words generated by the term frequency-inverse document frequency (TF-IDF) model to enhance semantic clarity and analytical accuracy. Specifically, high-frequency functional words (eg, “can,” “need”) that lacked substantive meaning, as well as unrecognized neologisms or colloquialisms that could distort topic clustering outcomes, were manually reviewed and removed.

This cleaning and standardization process was independently carried out by 2 researchers (HL and ZZ) with expertise in computational linguistics and health communication. Any discrepancies were resolved through cross-validation and iterative discussion, ensuring the reliability and reproducibility of subsequent semantic analyses.

### Public Medical Appeals Theme Identification

#### Feature Word Extraction Based on the TF-IDF Word Vector Model

In natural language processing, textual data are inherently unstructured and must be converted into a structured format interpretable by computational models [46]. Word vectorization offers a widely adopted solution by converting words and documents into mathematical representations (vectors) [47]. Over the years, several word vectorization techniques have been developed, including continuous bag of words, skip-gram, TF-IDF, and N-gram models [48-51].

Among these, the TF-IDF model offers distinct advantages over traditional bag-of-words approaches. It preserves the semantic representativeness of textual content while effectively reducing redundant feature dimensions, thereby improving both computational efficiency and model stability. Moreover, TF-IDF is particularly well-suited to short texts, such as social media posts, as it enhances the representation of localized semantic contexts and facilitates the modeling of unstructured information [52,53].

Accordingly, this study used the TF-IDF model to extract feature word weights and vectorize the public medical appeal dataset. Based on the ranked weights generated by the model, we identified and categorized core feature words for 3 primary types of appeals—suggestions, complaints/help-seeking, and consultations. A detailed list of keywords corresponding to each category is presented in Table S1 in Multimedia Appendix 1.

#### Feature Word Clustering Based on Systematic Clustering Algorithm

To identify the core themes of public medical appeals, this study applies a systematic clustering algorithm based on both a similarity matrix and a dissimilarity matrix derived from feature word co-occurrence data.

#### Construction of the Similarity Matrix

The similarity matrix was built using co-occurrence combinations of feature words. When 2 or more feature words frequently appear within the same text, they are assumed to exhibit semantic proximity. The number of co-occurrence connections reflects the relative importance and associative closeness of the terms, while the frequency of co-occurrence indicates the degree of correlation between them [54].

Among various normalization methods, the Ochiai coefficient is one of the most widely used in academic research for measuring co-occurrence strength. It is defined as follows [55,56]:


(1) $$\begin{array}{l} Ochiai\;coefficient=\left(The\;frequency\;of\;simultaneous\;occurrence\;of\;feature\;words\;AB\right)/\;\left(\sqrt{\left(The\;total\;frequency\;of\;occurrence\;of\;feature\;word\;A\right)}\times \;\sqrt{\left(The\;total\;frequency\;of\;occurrence\;of\;feature\;word\;B\right)}\right) \end{array}$$

This coefficient was used to construct the feature word similarity matrix, which captures the pairwise semantic affinity between terms (see Table S2 in Multimedia Appendix 1).

#### Construction of the Dissimilarity Matrix

To derive the dissimilarity matrix, each value of the Ochiai coefficient was subtracted from 1, thereby quantifying the semantic distance between pairs of feature words [57]:


(2) Dissimilarity=1−Ochiai Coefficient

This transformation ensures that dissimilarity values closer to 0 indicate stronger semantic associations between 2 keywords, whereas values approaching 1 indicate minimal or no association. The dissimilarity matrix for all feature words is presented in Table S3 in Multimedia Appendix 1.

#### Systematic Cluster Analysis

Using the dissimilarity matrix, a systematic cluster analysis was performed to identify thematic groupings within the public medical appeals dataset [58]. The clustering procedure used the intergroup linkage method and adopted Pearson correlation as the similarity criterion. A dendrogram was generated to visualize the hierarchical relationships among the resulting feature word clusters.

The resulting thematic clusters were then categorized based on core topic areas and encoded as categorical variables for subsequent regression analysis. This approach not only facilitates the integration of semantic themes into quantitative modeling but also provides a robust basis for examining how different appeal themes influence the likelihood of government response within the regression framework.

### Public Medical Appeals Sentiment Analysis

Sentiment analysis of textual data generally relies on 2 primary methodological approaches: machine learning–based models and lexicon-based methods. To enhance analytical robustness, this study adopts a dual-method strategy. The main empirical analysis is based on sentiment classifications generated through machine learning algorithms [59], while sentiment scores derived from a lexicon-based approach serve as a comparative reference [60].

For sentiment classification via machine learning, the study uses Python (Python Foundation) to interface with the natural language processing module of the Baidu AI Open Platform. This platform applies deep learning models to automatically identify sentiment-bearing words and analyze their contextual meanings. Each public medical appeal is classified as positive, neutral, or negative to provide an intuitive overview of sentiment orientation within the dataset (see Table S4 in Multimedia Appendix 1).

The natural language processing module of Baidu AI, a leading Chinese internet services and artificial intelligence technology company, enables fine-grained sentiment analysis by evaluating both the directionality (positive or negative) and the intensity of sentiment expressions. Leveraging advanced deep learning techniques, Baidu’s sentiment engine automatically learns complex semantic and syntactic structures, offering strong generalization capabilities and improved accuracy, particularly for longer and more context-rich sentences. This makes it significantly more effective than traditional lexicon-based sentiment analysis methods, which are often constrained by predefined word lists and static scoring mechanisms.

The resulting sentiment intensity scores generated via machine learning are subsequently incorporated into the regression model as independent variables, representing both the emotional valence and the strength of each appeal. This encoding approach allows the model to quantitatively account for the influence of emotional tone on the likelihood of government response.

### Constraining Factors in Government Online Responses to Public Medical Appeals

Drawing upon the research hypotheses proposed in this study, a binary logistic regression analysis was applied to model government online responses to public medical appeals. Two models were constructed. The first examines the influence of appeal text factors on government responses. The second builds on this by incorporating nontextual factors to explore the constraints on government responsiveness. The complete logistic regression model is represented as follows:


(3) $$\begin{array}{rl} \;logit\left(Y_{i}\right)=\; & \beta_{1}(ATH_{i})+\beta_{2}(AC_{i})+\beta_{3}(AE_{i})+\beta_{4}(ATI_{i})+\beta_{5}(DRI_{i})\\ +\beta_{6}(BA_{i})+\beta_{7}(EDL_{i})+\beta_{8}(IPD_{i})+\beta_{9}(ECL_{i})+\beta_{10}(LUD_{i})+E \end{array}$$

where $\beta_{1}$-$\beta_{4}$ represent the appeal theme, appeal content, appeal sentiment, and appeal title, respectively, among the demand text factors; $\beta_{5}$-$\beta_{10}$ represent the difficulty of resolving appeal incidents, benefit attribution, internet penetration, educational level, economic level, and disparities in labor union development, respectively, among the nontextual factors. The specific code scheme is provided in Table S5 in Multimedia Appendix 1.

To ensure the accuracy of the research results, this study conducted robustness tests on the factors influencing government online responses to public medical appeals. Specifically, probit regression analysis was applied to the sample data to test the robustness of the government response model.

## Results

### Data Filtering and Categorization

**Figure 1.**
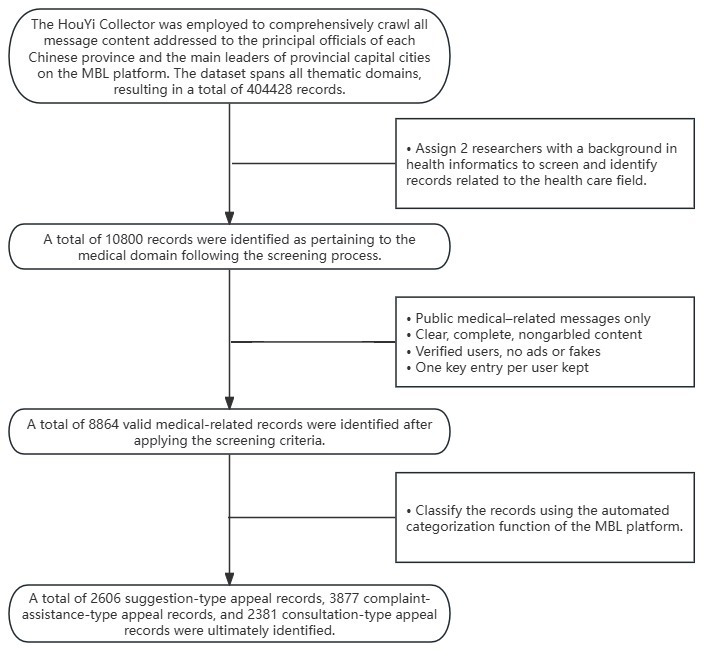
Flowchart of data filtering and categorization process. MBL: Message Board for Leaders.

As shown in Figure 1, after data cleaning and filtering, a total of 8864 valid medical appeal records were obtained, comprising 2606 suggestion-type appeals, 3877 complaint-assistance-type appeals, and 2381 consultation-type appeals.

### Public Medical Appeals Section

**Figure 2.**
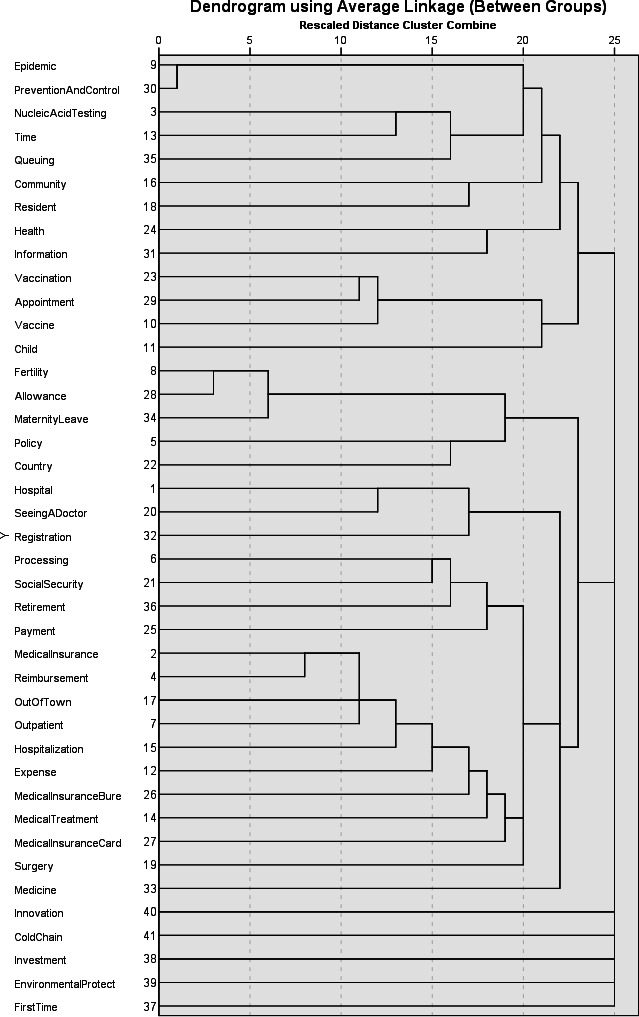
Results of the clustering analysis of themes in public medical appeals.

As shown in the clustering dendrogram (Figure 2), public medical appeals can be broadly divided into 5 major clusters at a distance of 23: (1) epidemic, prevention and control, nucleic acid testing, and time; (2) resident, health, information, and vaccine; (3) child, fertility, allowance, and maternity; (4) hospital, seeing a doctor, registration, and retirement; and (5) medical insurance card, surgery, and expense.

In the sentiment section of public medical appeals, the analysis demonstrates that applying different sentiment analysis methods can yield varying sentiment inclination results for the same set of public health care appeals. However, despite methodological differences, several common issues emerge. For instance, the analysis consistently shows that negative emotions are considerably more prevalent than positive emotions in the public medical appeals of Chinese citizens.

### Government’s Online Responses Section

#### Textual Factors and Government Responsiveness

As shown in Table 1 and Table S6 in Multimedia Appendix 1, the results of both logistic regression and probit regression models suggest that textual features, including the theme of the appeal, content complexity, emotional tone, and title structure, do not exhibit statistically significant associations with the likelihood of governmental response. Specifically, using *epidemic issue* as the reference category under the theme of the appeal, the *P* values are as follows: *fertility issue* (*P*=.63), *hospital issue* (*P*=.63), *security issue* (*P*=.72), and *other issues* (*P*=.34). Other textual features also show nonsignificant results, including *appeal content* (*P*=.80), *appeal sentiment* (*P*=.64), and *appeal title* (*P*=.55). Accordingly, hypotheses H1a, H1b, H1c, and H1d are not supported by the empirical data. This finding indicates that, within the context of the Chinese government’s online responses to public medical appeals, the textual characteristics of appeals play a relatively limited role in influencing administrative decision-making processes.

**Table 1.** Logistic regression results for factors influencing government responses to public medical appeals (n=8864).

Variables	Model 1				Model 2			
	Standardized regression coefficient (β)	SE	Exp(β) (95% CI)	*P* value	Standardized regression coefficient (β)	SE	Exp(β) (95% CI)	*P* value
Appeal theme								
Epidemic issue (reference)	N/A ^a^	N/A	N/A	N/A	N/A	N/A	N/A	N/A
Fertility issue	.058	0.163	1.060 (0.770-1.460)	.72	.084	0.173	1.087 (0.775-1.526)	.63
Hospital issue	.148	0.106	1.160 (0.942-1.427)	.16	.054	0.113	1.056 (0.846-1.317)	.63
Security issue	−.030	0.134	0.970 (0.746-1.262)	.82	−.052	0.144	0.949 (0.715-1.259)	.72
Other issue	.114	0.106	1.121 (0.911-1.380)	.28	.107	0.113	1.113 (0.892-1.388)	.34
Appeal content	4.201×10^−4^	6.917×10^−4^	1.000 (0.999-1.002)	.54	1.916×10^−4^	7.639×10^−4^	1.000 (0.998-1.001)	.80
Appeal sentiment	.228	0.235	1.256 (0.793-1.991)	.33	.117	0.248	1.124 (0.691-1.828)	.64
Appeal title	.001	0.006	1.001 (0.990-1.012)	.89	.004	0.006	1.004 (0.992-1.016)	.55
Difficulty of resolving incidents								
Low difficulty (reference)	N/A	N/A	N/A	N/A	N/A	N/A	N/A	N/A
Moderate difficulty	N/A	N/A	N/A	N/A	−.073	0.134	0.929 (0.715-1.208)	.59
High difficulty	N/A	N/A	N/A	N/A	.011	0.224	1.011 (0.652-1.568)	.96
Benefits attribution								
Individual interest (reference)	N/A	N/A	N/A	N/A	N/A	N/A	N/A	N/A
Collective interest	N/A	N/A	N/A	N/A	−.084	0.072	0.919 (0.798-1.059)	.25
Internet penetration degree								
Low internet area (reference)	N/A	N/A	N/A	N/A	N/A	N/A	N/A	N/A
Medium internet area	N/A	N/A	N/A	N/A	.456	0.158	1.577 (1.157-2.150)	.004
High internet area	N/A	N/A	N/A	N/A	2.296	0.174	9.930 (7.063-13.959)	<.001
Educational level								
Low education area (reference)	N/A	N/A	N/A	N/A	N/A	N/A	N/A	N/A
Medium education area	N/A	N/A	N/A	N/A	.915	0.122	2.497 (1.968-3.169)	<.001
High education area	N/A	N/A	N/A	N/A	−.107	0.130	0.899 (0.697-1.159)	.41
Economic level								
Low GDP^b^ area (reference)	N/A	N/A	N/A	N/A	N/A	N/A	N/A	N/A
Medium GDP area	N/A	N/A	N/A	N/A	.955	0.149	2.599 (1.941-3.479)	<.001
High GDP area	N/A	N/A	N/A	N/A	−.218	0.159	0.804 (0.589-1.098)	.17
Labor union development								
Low development area (reference)	N/A	N/A	N/A	N/A	N/A	N/A	N/A	N/A
Medium development area	N/A	N/A	N/A	N/A	−.571	0.145	0.565 (0.426-0.750)	<.001
High development area	N/A	N/A	N/A	N/A	−2.153	0.166	0.116 (0.084-0.161)	<.001

a N/A: not applicable.

b GDP: gross domestic product.

#### Transactional Factors and Government Responsiveness

As indicated in Table 1 and Table S6 in Multimedia Appendix 1, neither the difficulty of resolving the appeal nor the attribution of benefits shows a statistically significant association with the likelihood of a governmental online response. Specifically, regarding *difficulty of resolving incidents*, with *low difficulty* as the reference, *moderate difficulty* (*P*=.59) and *high difficulty* (*P*=.96) yield nonsignificant results. For *benefits attribution*, using *individual interest* as the baseline, *collective interest* also shows no significant association (*P*=.25). Accordingly, hypotheses H2a and H2b are not supported by the data. These findings suggest that, within the health care domain, governmental responsiveness on digital platforms is not substantially influenced by the transactional characteristics of public appeal.

#### Macro-Level Environmental Factors and Government Responsiveness

In contrast to textual and transactional factors, macro-level environmental variables, including internet penetration, education level, regional economic development, and labor union development, were found to significantly influence the likelihood of government responses to public medical appeals (Table 1).

Specifically, compared with regions with low internet penetration, appeals originating from medium- and high-penetration areas were significantly more likely to receive government responses, with odds ratios [Exp(β)] of 1.577 (95% CI 1.157-2.150, *P*=.004) and 9.930 (95% CI 7.063-13.959, *P*<.001), respectively. Using low-education regions as the reference group, appeals from medium-education regions had an odds ratio of 2.497 (95% CI 1.968-3.169, *P*<.001). Similarly, appeals from medium–gross domestic product regions showed an odds ratio of 2.599 (95% CI 1.941-3.479, *P*<.001) compared with those from low–gross domestic product regions.

By contrast, regions with more developed labor unions were associated with a lower likelihood of receiving government responses. Specifically, appeals from medium- and high-development labor union regions were only 0.565 times (95% CI 0.426-0.750, *P*<.001) and 0.116 times (95% CI 0.084-0.161, *P*<.001) as likely to receive responses, respectively, compared with regions with less developed labor unions.

Hypotheses H3a, H3b, H3c, and H3d are thus empirically supported. These findings underscore that, in the domain of public health care, governmental responsiveness on digital platforms is considerably shaped by broader macro-structural conditions, highlighting the importance of infrastructural and socioeconomic capacity in enabling effective digital governance.

## Discussion

### Principal Findings

#### From Sentiment to Structure: Explaining Government Response Patterns in Online Medical Appeals

This study investigated government online responses to public medical appeals. By integrating both textual and nontextual factors, it systematically examined the multidimensional mechanisms that influence governmental responsiveness. Compared with existing single-dimensional analytical strategies, the integrative model developed in this study broadens the explanatory scope for understanding how governments respond to public demands in the health care sector.

The key findings are as follows. First, the predominant themes of public medical appeals centered on pandemic prevention and control, fertility policy adjustments, access to medical care, and health insurance. Second, negative sentiment was overwhelmingly dominant across appeal types. Unlike social media platforms that often serve as spaces for general discussion or emotional venting, the MBL—with its strong orientation toward problem-solving—is perceived by the public as an effective channel for addressing concrete medical grievances and influencing health policy reform. Third, government responsiveness to public medical appeals was found to be significantly influenced by macro-level environmental factors, including internet penetration, education level, economic development, and labor union development. These findings challenge conventional hypotheses suggesting that linguistic expression or appeal content alone determines responsiveness, instead highlighting the relative indifference or neutrality of governmental response mechanisms in the health care sector with respect to individual textual features.

#### Public Medical Appeals Section

In the analysis of appeal themes, it is evident that over the past 2 years, public health care demands in China have been significantly shaped by emergent public health events, particularly the COVID-19 pandemic, leading to stronger and more urgent expressions of public needs. Fertility issues have also emerged as a major area of concern. In response to structural demographic shifts, the Chinese government has continuously adjusted its fertility policies, which directly influence reproductive intentions and behaviors at the household level [61]. Furthermore, the efficiency of hospital services and the effectiveness of disease treatment remain central to the broader social issue known as *Kan Bing Gui,* which refers to the difficulty and high cost associated with accessing medical care in China. Cross-regional medical treatment and reimbursement challenges under the national basic medical insurance system also continue to be major focal points of public concern.

In the analysis of sentiment embedded in public medical appeals, negative emotions were found to significantly outweigh positive ones. This pattern reflects widespread public apprehension and indicates that health care**–**related demands remain largely unmet. The predominance of negative sentiment suggests a misalignment between the public’s expectations and the current capacity of China’s health care service system. This discrepancy underscores the urgent need to improve the accessibility, equity, and quality of health care services to alleviate public dissatisfaction and emotional distress.

#### Government’s Online Responses Section

With respect to internet penetration, a higher degree of penetration was found to be directly proportional to the likelihood of government online responsiveness. Greater internet penetration facilitates broader access to and dissemination of information, enabling the public to better understand the quality of medical services and the availability of resources. It also provides a more convenient and efficient channel for different sectors of society to raise concerns, thereby allowing medical issues to be surfaced and addressed more promptly.

In terms of education level, residents in well-educated regions are more likely to possess a broader and deeper knowledge base, contributing to overall improvements in individual capacity. The improvement of overall individual competence enhances political awareness and participatory capacity, enabling the public to engage more effectively and proactively in the political process and to express personal appeals more reasonably on online governance platforms. Additionally, the enrichment of educational resources provides strong support for cultivating professional medical talents, promoting medical research, and fostering innovation in medical services.

In terms of economic development level, people in economically developed provinces are more inclined to express their medical concerns. These areas often have more complete and advanced medical systems, enabling the public to advocate more confidently for their rights and demands in medical matters. Consequently, individuals in economically developed regions tend to place greater emphasis on personal health and are more likely to voice their medical needs, expecting the health care system to better accommodate individual differences and special requirements.

In terms of labor union development, the maturity and organizational activity of trade unions also exert a significant influence on government responsiveness. These institutions not only articulate collective concerns and monitor policy implementation more systematically but also help establish feedback mechanisms within the governance process. By fulfilling co-governance and co-construction functions, trade unions may alleviate pressure on governments to respond directly. Therefore, in regions where trade unions are more institutionalized and actively engaged, the frequency of individual-level government responses may be comparatively lower—not due to a lack of attention, but because some responsive functions have been effectively assumed by the unions themselves.

### Comparison With Prior Work

This study found that textual factors do not have a significant impact on government responses. This challenges much of the prior literature, which generally argues that the linguistic and emotional strategies used in appeals can influence governmental responsiveness motivations [28,62-64]. However, some divergent findings have emerged. A field experiment covering 140 “Mayor’s Mailboxes” across China showed that variations in citizens’ discourse styles did not significantly affect either the likelihood or the speed of government responses [23]. Similarly, Zhang et al [65] found that the length of appeals had only a weak effect on government responsiveness, and that government reactions were not simply driven by the emotional tone of appeals, reflecting a relatively high level of procedural maturity in official response mechanisms.

In addition, this study found that nontextual factors, including benefit attribution and the perceived difficulty of incident resolution, did not exhibit statistically significant effects on government responsiveness. This suggests that, within the domain of public medical appeals, the government’s response mechanism may operate independently of specific issue characteristics. Such a finding indicates a relatively stable and standardized pattern of responsiveness, potentially reflecting institutionalized response protocols or normative governance practices in the digital public service context. This finding is closely linked to the unique characteristics of health care governance in China. In Chinese culture, it is often said that “health care bears no trivial matters, and the responsibility is as heavy as a mountain,” highlighting the immense societal importance attributed to medical services. The universalistic nature of health care services means that governments adhere to principles of fairness and equality, ensuring that all citizens’ health care needs—regardless of scale—receive appropriate attention and action. This policy direction not only reflects the inclusiveness of public services but also helps promote social equity and harmony, thereby enhancing the coverage and accessibility of medical care.

Moreover, as China has sought to institutionalize information disclosure, consultative democracy, and digital democracy, institutionalized normative review mechanisms have emerged that mandate government responsiveness. These arrangements aim to minimize political risk and ensure timely, continuous government engagement with citizen appeals [11,66].

Thus, in the health care domain, neither textual nor event-related factors emerge as significant determinants of government responses. Instead, macro-environmental factors, such as economic development, education levels, internet penetration rates, and labor union development, exert a far greater influence. This finding aligns with prior studies by Zhao et al [67], Hu et al [68], and Gauld et al [69]. Accordingly, policy makers should adjust digital governance frameworks to prioritize macro-environmental conditions in shaping governmental responsiveness. Specifically, governments should develop seamless e-government systems that enable cross-regional, cross-organizational, and cross-sectoral coordination. Such systems would help to identify and bridge complex public needs arising from disparities in economic, educational, technological, and social development across regions. Furthermore, response mechanisms should be designed with sufficient flexibility, and digital governance platforms should leverage big data analytics to integrate and analyze macro-environmental factors. This would allow governments to dynamically adjust response strategies in the face of changing economic conditions or public health emergencies, thereby ensuring fairness in government responsiveness.

Previous studies have also found that during sudden public crises, routine governance activities and resource allocation decisions are adjusted based on crisis dynamics, and government responsiveness exhibits distinct features [7,11,70]. This study spans a critical transitional period in China’s pandemic control policy; as a result, public medical appeal sentiments were significantly influenced by the COVID-19 public health emergency.

Sentiment analysis revealed that negative emotions far outweighed positive ones, reaching a substantial proportion (6704/8864, 75.63%). This proportion is significantly higher than findings from several studies that assessed public sentiment during the pandemic using social media data sources [71-73]. On platforms such as *Weibo*, *Twitter*, and *Facebook*, public sentiment is generally more balanced and, in some cases, even predominantly positive. For example, one study reported that 72.9% of evaluations of nucleic acid testing policies were positive [71], while discussions surrounding vaccines were often characterized by trust and optimism [72].

This divergence in findings may be attributed to functional differences between platforms. Social media platforms such as *Twitter* and *Facebook* serve primarily as spaces for public discourse, emotional expression, and opinion oversight [8,74,75]. By contrast, the MBL—a government-led appeal feedback channel—has a stronger problem-solving orientation. The high prevalence of negative sentiment on this platform closely correlates with the exposure of real, unresolved problems, as individuals tend to voice concrete grievances rather than engage in generalized discussion. The public’s preference for expressing concerns through official digital platforms rather than commercial social media suggests a degree of trust in the government’s capacity to intervene effectively.

### Strengths and Limitations

This study offers several notable strengths. First, unlike previous research that primarily utilized data from general online governance platforms and often lacked thematic specificity, this study focuses explicitly on government responsiveness in the health care sector. It notably reveals that variations in textual features or public discourse styles do not lead to differentiated government responses to health care**–**related public appeals.

Second, while prior studies have often examined fragmented textual or nontextual factors in isolation, this study proposes research hypotheses that integrate both dimensions. This comprehensive strategy opens the “black box” of government responsiveness mechanisms, offfering a systematic and holistic perspective on how different factors interact to shape online public service responses, particularly within the sensitive and complex domain of healthcare governance.

Third, the study reveals the thematic structure, emotional tone, and governmental response logic of public health care**–**related appeals. These findings contribute to optimizing government response mechanisms on digital health platforms, improving response rates, and providing novel theoretical and empirical insights into the dynamics of health information dissemination in the digital era. In doing so, the study enriches ongoing practices in digital health governance.

Nonetheless, several limitations should be acknowledged.

One major limitation concerns potential sample selection bias. As the MBL is an online platform, the study may inadvertently exclude older adults and individuals with lower socioeconomic status or limited digital literacy—populations that are also highly relevant in the context of health care demands.

Another limitation lies in the use of the TF-IDF algorithm. While it remains a widely accepted method for feature extraction, TF-IDF does not account for word order or deeper semantic relationships between terms. As a result, the contextual meanings embedded in public medical appeals may not be fully captured, potentially affecting the accuracy of theme interpretation.

Additionally, although the study considers both textual and nontextual factors, it incorporates only 4 macro-environmental variables—economic development, education level, internet penetration rate, and labor union development—to represent external structural influences. This limited variable set may constrain the breadth and depth of the study’s explanatory capacity, leaving out other potentially influential factors such as local governance capacity or health system maturity.

### Future Directions

To address the issue of sample selection bias, future research could adopt a mixed-methods approach that combines online data mining with offline data collection techniques, such as semistructured interviews or questionnaire surveys. This would enable researchers to obtain firsthand data from underrepresented populations, such as older adults with limited digital access, thereby enhancing sample representativeness and inclusivity.

From a methodological standpoint, future studies may consider improving text analysis accuracy by integrating TF-IDF with semantic embedding techniques, such as Word2Vec or GloVe. These models can more effectively capture semantic proximity and contextual nuance, providing a more refined understanding of the emotional and thematic content in public appeals.

Moreover, to expand the explanatory power of the macro-structural dimension, future research should incorporate additional macro-environmental variables, such as regional levels of e-government development and the scale of public financial investment in health care. Including such factors would allow for a more robust interpretation of how institutional and structural contexts shape government responsiveness in digital health governance.

### Conclusions

Government responsiveness to public appeals through online platforms embodies a core tenet of New Public Management, serving as a vital mechanism for strengthening modern governance systems and enhancing administrative capacity. In the context of health care, such responsiveness not only enables more agile and adaptive policy implementation but also serves as a strategic pathway for optimizing health care service delivery. By facilitating real-time feedback and public engagement, digital responsiveness reshapes the logic and practices of governance in the health sector, fostering greater institutional resilience and citizen trust.

Drawing on large-scale textual data from the MBL, this study employed a combination of TF-IDF feature extraction, sentiment analysis, and logistic regression to identify thematic patterns and emotional characteristics embedded in public medical appeals submitted via China’s digital governance platforms. By systematically integrating both textual and nontextual dimensions, this study uncovered and empirically validated the multidimensional mechanisms that shape government responsiveness to public appeals in the health care sector.

The findings indicate that public medical appeals primarily cluster around topics such as pandemic control, fertility policies, health care accessibility, and medical insurance, with a general predominance of negative sentiment expression. Contrary to conventional hypotheses that emphasize the role of textual features, such as linguistic tone or emotional intensity, this study finds that the salience of specific language features and issue types has a limited impact on response likelihood within the health care domain. Instead, macro-environmental variables, including regional levels of education, internet penetration, economic development, and labor union development, significantly influence the probability of receiving a governmental response. These results reflect the complex interplay between issue sensitivity, bureaucratic discretion, and political considerations in shaping institutional response behavior.

In sum, this research contributes both theoretically and empirically to the understanding of government responsiveness in the digital age, especially within high-stakes sectors such as health care. It offers transferable empirical strategies for scholars and practitioners aiming to evaluate and enhance responsive governance across other domains of public service delivery. In doing so, it informs ongoing efforts to modernize digital government and advance citizen-centered governance paradigms.

## Supplementary material

10.2196/70087 Multimedia Appendix 1 Additional analysis.

## Abbreviations

MBL: Message Board for Leaders
mHealth: mobile health
TF-IDF: term frequency-inverse document frequency
