# Supplementary material for: Public Medical Appeals and Government Online Responses: Big Data Analysis Based on Chinese Digital Governance Platforms
Source: J Med Internet Res. 2025 Aug 6;27:e70087. doi: 10.2196/70087 (PMC12327965; doi:10.2196/70087)
Supplement: Multimedia Appendix 1 [file jmir-v27-e70087-s001.docx]

**Table S1.** Extracted Feature Words from Public Medical Appeals

|  | Suggestion-Type Appeal Records | Complaint-Assistance-Type Appeal Records | Consultation-Type Appeal Records |
| --- | --- | --- | --- |
|  |  |  |  |
| 1 | First time[第一时间] | Hospital[医院] | Time[时间] |
|  |  |  |  |
| 2 | Fault[故障] | Medical insurance  [医保] | Investment[投资] |
|  |  |  |  |
| 3 | Cold chain[冷链] | Reimbursement[报销] | Innovation[创新] |
|  |  |  |  |
| 4 | Hospital[医院] | Outpatient[门诊] | Environmental protection[环保] |
|  |  |  |  |
| 5 | Medical insurance[医保] | Hospitalization[住院] | Medical insurance[医保] |
|  |  |  |  |
| 6 | Nucleic acid testing[核酸检测] | Expense[费用] | Hospital[医院] |
|  |  |  |  |
| 7 | Epidemic[疫情] | Vaccine[疫苗] | Reimbursement[报销] |
|  |  |  |  |
| 8 | Reimbursement[报销] | Surgery[手术] | Policy[政策] |
|  |  |  |  |
| 9 | Policy[政策] | Social security[社保] | Fertility[声誉] |
|  |  |  |  |
| 10 | Vaccine[疫苗] | Medical Insurance Bureau[医保局] | Processing[办理] |
|  |  |  |  |
| 11 | Outpatient[门诊] | Fertility[生育] | Outpatient[门诊] |
|  |  |  |  |
| 12 | Fertility[生育] | Policy[政策] | Vaccine[疫苗] |
|  |  |  |  |
| 13 | Prevention and control[防控] | Payment[缴费] | Expense[费用] |
|  |  |  |  |
| 14 | Community[社区] | Nucleic acid testing[核酸检测] | Out-of-town[异地] |
|  |  |  |  |
| 15 | Medical treatment[医疗] | Community[社区] | Vaccination[接种] |
|  |  |  |  |
| 16 | Health[健康] | Epidemic[疫情] | Child[孩子] |
|  |  |  |  |
| 17 | Expense[费用] | Medical insurance card[医保卡] | Resident[居民] |
|  |  |  |  |
| 18 | Seeing a doctor[看病] | Registration[挂号] | Country[国家] |
|  |  |  |  |
| 19 | Hospitalization[住院] | Information[信息] | Allowance[津贴] |
|  |  |  |  |
| 20 | Out-of-town[异地] | Allowance[津贴] | Epidemic[疫情] |
|  |  |  |  |
| 21 | Queueing[排队] | Health[健康] | Social security[社保] |
|  |  |  |  |
| 22 | Appointment[预约] | Retirement[退休] | Maternity leave[产假] |
|  |  |  |  |
| 23 | Vaccination[接种] | Medicine[药品] | Nucleic acid testing[核酸检测] |
|  |  |  |  |

**Table S2**. Similarity Matrix of Extracted Feature Words from Public Medical Appeals

|  | | | Hospital | | | Medical insurance | | | Nucleic acid testing | | | Reimbursement | | | Policy | | | Processing | | Outpatient | | |
| --- | --- | --- | --- | --- | --- | --- | --- | --- | --- | --- | --- | --- | --- | --- | --- | --- | --- | --- | --- | --- | --- | --- |
|  |  |  | |  |  | |  |  | |  |  | |  |  | |  |  | |  | |  |  |
| Hospital | | | 1.000 | | | 0.368 | | | 0.229 | | | 0.431 | | | 0.120 | | | 0.226 | | 0.483 | | |
|  | | |  | | |  | | |  | | |  | | |  | | |  | |  | | |
| Medical insurance | | | 0.368 | | | 1.000 | | | 0.004 | | | 1.000 | | | 0.443 | | | 0.625 | | 0.519 | | |
|  | | |  | | |  | | |  | | |  | | |  | | |  | |  | | |
| Nucleic acid testing | | | 0.229 | | | 0.004 | | | 1.000 | | | 0.001 | | | 0.160 | | | 0.016 | | 0.046 | | |
|  | | |  | | |  | | |  | | |  | | |  | | |  | |  | | |
| Reimbursement | | | 0.431 | | | 1.000 | | | 0.001 | | | 1.000 | | | 0.331 | | | 0.223 | | 0.705 | | |
|  | | |  | | |  | | |  | | |  | | |  | | |  | |  | | |
| Policy | | | 0.120 | | | 0.443 | | | 0.160 | | | 0.331 | | | 1.000 | | | 0.118 | | 0.189 | | |
|  | | |  | | |  | | |  | | |  | | |  | | |  | |  | | |
| Processing | | | 0.226 | | | 0.625 | | | 0.016 | | | 0.223 | | | 0.118 | | | 1.000 | | 0.146 | | |
|  | | |  | | |  | | |  | | |  | | |  | | |  | |  | | |
| Outpatient | | | 0.483 | | | 0.519 | | | 0.046 | | | 0.705 | | | 0.189 | | | 0.146 | | 1.000 | | |
|  | | |  | | |  | | |  | | |  | | |  | | |  | |  | | |
| Fertility | | | 0.065 | | | 0.301 | | | 0.000 | | | 0.425 | | | 0.442 | | | 0.228 | | 0.011 | | |
|  | | |  | | |  | | |  | | |  | | |  | | |  | |  | | |
| Epidemic | | | 0.154 | | | 0.052 | | | 0.536 | | | 0.043 | | | 0.227 | | | 0.087 | | 0.043 | | |
|  | | |  | | |  | | |  | | |  | | |  | | |  | |  | | |
| Vaccine | | | 0.190 | | | 0.023 | | | 0.024 | | | 0.008 | | | 0.047 | | | 0.038 | | 0.032 | | |
|  | | |  | | |  | | |  | | |  | | |  | | |  | |  | | |
| Child | | | 0.323 | | | 0.317 | | | 0.112 | | | 0.232 | | | 0.151 | | | 0.234 | | 0.139 | | |
| …… | | | …… | | | …… | | | …… | | | …… | | | …… | | | …… | | …… | | |
|  | | |  | | |  | | |  | | |  | | |  | | |  | |  | | |

*Note*: Only partial results are presented.

**Table S3**. Partial Dissimilarity Matrix of Feature Terms from Public Medical Appeals

|  | Hospital | Medical insurance | Nucleic acid testing | Reimbursement | Policy | Processing | Outpatient |
| --- | --- | --- | --- | --- | --- | --- | --- |
|  |  |  |  |  |  |  |  |
| Hospital | 0.000 | 0.632 | 0.771 | 0.569 | 0.880 | 0.774 | 0.517 |
|  |  |  |  |  |  |  |  |
| Medical insurance | 0.632 | 0.000 | 0.996 | 0.000 | 0.557 | 0.375 | 0.481 |
|  |  |  |  |  |  |  |  |
| Nucleic acid testing | 0.771 | 0.996 | 0.000 | 0.999 | 0.840 | 0.984 | 0.954 |
|  |  |  |  |  |  |  |  |
| Reimbursement | 0.569 | 0.000 | 0.999 | 0.000 | 0.669 | 0.777 | 0.295 |
|  |  |  |  |  |  |  |  |
| Policy | 0.880 | 0.557 | 0.840 | 0.669 | 0.000 | 0.882 | 0.811 |
|  |  |  |  |  |  |  |  |
| Processing | 0.774 | 0.375 | 0.984 | 0.777 | 0.882 | 0.000 | 0.854 |
|  |  |  |  |  |  |  |  |
| Outpatient | 0.517 | 0.481 | 0.954 | 0.295 | 0.811 | 0.854 | 0.000 |
|  |  |  |  |  |  |  |  |
| Fertility | 0.935 | 0.699 | 1.000 | 0.575 | 0.558 | 0.772 | 0.989 |
|  |  |  |  |  |  |  |  |
| Epidemic | 0.846 | 0.948 | 0.464 | 0.957 | 0.773 | 0.913 | 0.957 |
|  |  |  |  |  |  |  |  |
| Vaccine | 0.810 | 0.977 | 0.976 | 0.992 | 0.953 | 0.962 | 0.968 |
|  |  |  |  |  |  |  |  |
| Child | 0.677 | 0.683 | 0.888 | 0.768 | 0.849 | 0.766 | 0.861 |
| …… | …… | …… | …… | …… | …… | …… | …… |
|  |  |  |  |  |  |  |  |

*Note*: Only partial results are presented.

**Table S4.** Sentiment Analysis of Public Medical Appeals

| Method | Positive,  n (%)^a^ | Neutral,  n (%)^a^ | Negative,  n (%)^a^ | Mean Sentiment Intensity | Mean Sentiment Score |
| --- | --- | --- | --- | --- | --- |
| **Machine learning method** |  |  |  |  |  |
|  |  |  |  |  |  |
| suggestion-type  (n=2606) | 773(29.662) | 123(4.720) | 1710(65.618) | 0.877 | __^b^ |
|  |  |  |  |  |  |
| complaint-assistance-type  (n=3877) | 413(10.653) | 136(3.508) | 3328(85.840) | 0.924 | __ |
|  |  |  |  |  |  |
| consultation-type  (n=2381) | 549(23.058) | 166(6.972) | 1666(69.971) | 0.806 | __ |
|  |  |  |  |  |  |
| **Sentiment lexicon method** |  |  |  |  |  |
|  |  |  |  |  |  |
| suggestion-type  (n=2606) | 674(25.863) | 545(20.913) | 1387(53.223) | __ | 2.289 |
|  |  |  |  |  |  |
| complaint-assistance-type  (n=3877) | 591(15.244) | 649(16.740) | 2637(68.017) | __ | 0.277 |
|  |  |  |  |  |  |
| consultation-type  (n=2381) | 861(36.161) | 529(22.218) | 991(41.621) | __ | 1.977 |
|  |  |  |  |  |  |

*Note:* (a) Percentages have been rounded and may not total to 100%; (b) Not applicable.

**Table S5.** Definition and Coding of Independent Variables

| Variable | Symbol | Variable Description |
| --- | --- | --- |
|  |  |  |
| Government response | GR | 0=Not processed/In progress, 1=Processed |
|  |  |  |
| Appeal Theme | ATH | 1=Epidemic issue, 2=Fertility issue, 3=Hospital issue, 4=Security issue, 5=Other issue |
|  |  |  |
| Appeal Content | AC | LEN（Actual number of words in the appeal content） |
|  |  |  |
| Appeal sentiment | AE | Encoding based on the actual intensity of sentimental inclination |
|  |  |  |
| Appeal title | ATI | LEN（Actual number of words in the appeal title） |
|  |  |  |
| Difficulty of resolving incidents | DRI | 1=Low difficulty, 2=Moderate difficulty, 3=High difficulty |
|  |  |  |
| Benefits attribution | BA | 1=Individual interest, 2=Collective interest |
|  |  |  |
| Educational level | EDL | 1=Low education area, 2=Medium education area, 3=High education area^a^ |
|  |  |  |
| Internet penetration degree | IPD | 1=Low internet penetration area, 2=Medium internet penetration area, 3=High internet penetration area^b^ |
|  |  |  |
| Economic level | ECL | 1=Low GDP area, 2=Medium GDP area, 3=High GDP area^b^ |
|  |  |  |
| Labor union development | LUD | 1=Low development area, 2= Medium development area,3= High development area^b^ |

*Note:* (a) Data are based on the number of administrative units reported in the China Social Statistics Yearbook 2023; (b) Data are based on the China Statistical Yearbook 2023.

**Table S6.** Probit Logistic Regression Results for Factors Influencing Government Responses to Public Medical Appeals(n=8,864)

| Variables | Model 1 | | | | Model 2 | | | | |
| --- | --- | --- | --- | --- | --- | --- | --- | --- | --- |
|  |  | | |  |  |  |  |  | |
|  | β^a^ | SE | *z* score | *P* value | β | SE | *z* score | *P* value | |
|  |  |  |  |  |  |  |  |  | |
| **ATH** |  |  |  |  |  |  |  |  | |
| Epidemic issue (reference) | N/A^b^ | N/A | N/A | N/A | N/A | N/A | N/A | N/A | |
| Fertility issue | 0.031 | 0.088 | 0.348 | .728 | 0.021 | 0.093 | 0.222 | .824 | |
| Hospital issue | 0.079 | 0.057 | 1.386 | .166 | 0.017 | 0.061 | 0.283 | .777 | |
| Security issue | -0.017 | 0.073 | -0.230 | .818 | -0.032 | 0.079 | -0.409 | .683 | |
| Other issue | 0.061 | 0.057 | 1.065 | .287 | 0.049 | 0.061 | 0.804 | .421 | |
|  |  |  |  |  |  |  |  |  | |
| **AC** | 2.145×10  ^−4^ | 3.698×10  ^−4^ | 0.580 | .562 | −9.540×10  ^−5^ | 4.093×10  ^−4^ | -0.233 | .816 | |
|  |  |  |  |  |  |  |  |  |  |
|  |  |  |  |  |  |  |  |  | |
| **AE** | 0.123 | 0.126 | 0.977 | .328 | 0.072 | 0.133 | 0.541 | .589 | |
|  |  |  |  |  |  |  |  |  |  |
|  |  |  |  |  |  |  |  |  | |
| **ATI** | 4.284×10  ^−4^ | 0.003 | 0.139 | .889 | 0.002 | 0.003 | 0.678 | .498 | |
|  |  |  |  |  |  |  |  |  |  |
|  |  |  |  |  |  |  |  |  | |
| **DRI** |  |  |  |  |  |  |  |  | |
| Low difficulty (reference) | N/A | N/A | N/A | N/A | N/A | N/A | N/A | N/A | |
| Moderate difficulty | N/A | N/A | N/A | N/A | -0.021 | 0.073 | -0.292 | .770 | |
| High difficulty | N/A | N/A | N/A | N/A | 0.016 | 0.121 | 0.130 | .897 | |
|  |  |  |  |  |  |  |  |  | |
| **BA** |  |  |  |  |  |  |  |  | |
| Individual interest (reference) | N/A | N/A | N/A | N/A | N/A | N/A | N/A | N/A | |
| Collective interest | N/A | N/A | N/A | N/A | -0.047 | 0.039 | -1.206 | .228 | |
|  |  |  |  |  |  |  |  |  | |
| **IPD** |  |  |  |  |  |  |  |  | |
| Low internet area (reference) | N/A | N/A | N/A | N/A | N/A | N/A | N/A | N/A | |
| Medium internet area | N/A | N/A | N/A | N/A | 0.241 | 0.090 | 2.695 | .007 | |
| High internet  area | N/A | N/A | N/A | N/A | 1.215 | 0.096 | 12.595 | <.001 | |
|  |  |  |  |  |  |  |  |  |  |
|  |  |  |  |  |  |  |  |  | |
| **EDL** |  |  |  |  |  |  |  |  |  |
| Low education area (reference) | N/A | N/A | N/A | N/A | N/A | N/A | N/A | N/A | |
| Medium education area | N/A | N/A | N/A | N/A | 0.418 | 0.061 | 6.894 | <.001 | |
| High education area | N/A | N/A | N/A | N/A | -0.066 | 0.067 | -0.993 | .321 | |
|  |  |  |  |  |  |  |  |  | |
| **ECL** |  |  |  |  |  |  |  |  | |
| Low GDP area (reference) | N/A | N/A | N/A | N/A | N/A | N/A | N/A | N/A | |
| Medium GDP area | N/A | N/A | N/A | N/A | 0.514 | 0.077 | 6.693 | <.001 | |
| High GDP area | N/A | N/A | N/A | N/A | -0.045 | 0.083 | -0.533 | .594 | |
|  |  |  |  |  |  |  |  |  | |
| **LUD** |  |  |  |  |  |  |  |  | |
| Low development area (reference) | N/A | N/A | N/A | N/A | N/A | N/A | N/A | N/A | |
| Medium development area | N/A | N/A | N/A | N/A | -0.382 | 0.083 | -4.581 | <.001 | |
| High development area | N/A | N/A | N/A | N/A | -1.194 | 0.093 | -12.773 | <.001 | |
|  |  |  |  |  |  |  |  |  | |

*Note:* (a)Standardized regression coefficients ; (b) N/A: not applicable
